# Development of a versatile sample preparation method and its application for rare-earth pattern and Nd isotope ratio analysis in nuclear forensics

**DOI:** 10.1007/s10967-014-3505-3

**Published:** 2014-09-11

**Authors:** J. Krajko, Z. Varga, M. Wallenius, K. Mayer

**Affiliations:** 1 European Commission, Joint Research Centre, Institute for Transuranium Elements, Postfach 2340, 76125 Karlsruhe, Germany; 2 Faculty of Applied Sciences, Delft University of Technology, Mekelweg 15, 2629 JB Delft, Netherlands

**Keywords:** Nuclear forensics, Uranium ore concentrate, Lanthanides, Pre-concentration, Neodymium isotope ratio

## Abstract

An improved sample preparation procedure for trace-levels of lanthanides in uranium-bearing samples was developed. The method involves a simple co-precipitation using Fe(III) carrier in ammonium carbonate medium to remove the uranium matrix. The procedure is an effective initial pre-concentration step for the subsequent extraction chromatographic separations. The applicability of the method was demonstrated by the measurement of REE pattern and ^143^Nd/^144^Nd isotope ratio in uranium ore concentrate samples.

## Introduction

Since the beginning of the 1990s cases of illicit trafficking and other unauthorised acts involving nuclear material from various stages of the nuclear fuel cycle have taken place. Due to these incidents nuclear materials were started to be analysed in the context of criminal investigations, and a new branch of forensic science—nuclear forensics—was born. The IAEA defines the nuclear forensics as “the analysis of intercepted illicit nuclear or radioactive material and any associated material to provide evidence for nuclear attribution” [1]. The aim for nuclear forensic scientists is therefore to identify indicators which represent relationships between measurable parameters of the material and its production history.

These nuclear forensic indicators, so-called *signatures*, can be various properties of the material in question, such as structure, morphology, major and minor elements, isotopes and impurities. Among these properties there are only few exclusive parameters, which would give straightforward information about an unknown sample without the need to use in interpretation comparison samples or data. For example, the enrichment and concentration of uranium (U) of an investigated sample could lead us easily to the intended use of the sample or to the stage of the nuclear fuel-cycle from which the sample originates. However, the majority of the nuclear signatures belong to the so-called non-exclusive, comparative parameters. This means that the analytical results have to be compared with those of measured for known samples or reference data in databases in order to draw conclusions about the possible origin of the sample.

In order to support the investigations of unknown seized nuclear materials, besides the new method developments, the improvement of existing methodologies is important as well. Up to now the isotopic patterns of O, S, Pb, Sr, and U have been investigated and found to be valuable signatures [2–6]. Besides these signatures the ^143^Nd/^144^Nd isotope ratio was investigated recently, which is commonly used in geology for chronometry and provenance measurements. It was found a promising candidate for a new nuclear forensic signature, since the ratio is indicative of the age and types of the minerals present [7, 8]. Although the ^143^Nd/^144^Nd isotope ratio in most of the uranium ore concentrates (UOC) samples was possible to be measured with a sufficiently low uncertainty (~0.05 % RSD) there were still a few samples which contained so small amounts of Nd that the measurement was not possible with the standard method (i.e. TRU extraction chromatography) or it could be performed only with too large uncertainty [9].

This work presents an improved procedure developed for trace-level analysis of ^143^Nd/^144^Nd isotope ratio in UOC samples by inductively coupled plasma mass spectrometry (ICP-MS). The aim of the study was to develop an effective pre-concentration method prior to the chromatographic separations, which enables the measurement of Nd isotope ratio in uranium samples. Co-precipitation as the most effective pre-concentration method was selected to achieve the required limits of detection in the low pg g^−1^ range. For high-purity uranium materials the major challenge is to achieve a high separation factor from uranium. The removal of uranium is of necessity as relatively large amounts of samples (100–500 mg of U) are required to yield measurable quantities of the analytes, i.e., lanthanides (Ln). For such sample quantities the standard methods (e.g. direct extraction chromatography separation) cannot be applied, since the high amount of U precludes their use. Applying co-precipitation for the preconcentration of traces of Ln from larger amounts of environmental samples (e.g. sea water [10–12], geological samples [13, 14]) has been studied and it has been proved to be an effective method. Our procedure involves a co-precipitation of rare-earth elements as Fe(OH)_3_ in the presence of Fe(III) carrier, followed by an extraction chromatographic group separation of Ln and a sequential separation of Nd, Sm, and other heavy lanthanides. Though the primary purpose of the study is the separation of Ln, the methodology can be extended for the pre-concentration of other important elements for nuclear forensics present at trace-levels, such as Th, Am or Pu.

## Experimental

### Reagents

Thorough cleaning of all labware is necessary before use for trace-level measurement of Nd isotope ratio. This was performed with dilute ethanol, followed by dilute nitric acid, and finally with high purity water rinsing. For all the dilutions high-purity water was used (UHQ System, USF Elga, Germany). Hydrochloric and nitric acids were of Suprapur grade (Merck, Darmstadt, Germany), although the nitric acid was further purified by sub-boiling distillation.

Analytical grade Fe(NO_3_)_3_ salt was used as carrier for the co-precipitation (Alfa Aesar, Karlsruhe, Germany). Analytical grade sodium-hydroxide and ammonium-carbonate used for the precipitation were purchased from Sigma Aldrich (St Louis, MO, USA). Ammonium carbonate was further purified prior the use by adding about 10 mg of Fe^3+^ and precipitating Fe(OH)_3_ to remove the trace-level lanthanide impurities still present in the analytical grade ammonium carbonate solution.

For the lanthanide group separation, the TRU™ extraction chromatographic resin supplied by Triskem (Triskem International, Bruz, France) was used. For the preparation of columns, 1.6 mL of the resin was placed in plastic Bio-Rad holders (diameter 8 mm) and plugged with porous Teflon frit (Reichelt Chemietechnik Heidelberg, Germany) on the top of the resin to avoid mixing. For Nd separation, the Ln Resin™ for the extraction chromatographic separation was purchased from Triskem (Triskem International, Bruz, France). For the preparation of columns, 400 µL of the resin was placed in plastic Bio-Rad holders and plugged with porous Teflon frit.

For the optimization of the chemical separation procedure and the measurements by ICP-MS, lanthanide standard solution and monoelemental Nd and Sm standard solutions (Alfa Aesar, Karlsruhe, Germany) were prepared by the dilution from 1,000 to 100 μg mL^−1^ standard solutions, respectively. The U_3_O_8_ certified reference material, Morille (Cetama, France) was used for the validation of the co-precipitation method as it is certified for four lanthanides’ content (Dy, Gd, Eu and Sm).

### Instrumentation

The mass spectrometric analysis of aliquots from the co-precipitation step was carried out using an ELEMENT2 (Thermo Electron Corp., Bremen, Germany) double-focusing magnetic sector inductively coupled plasma mass spectrometer (ICP-SFMS). Measurements were carried out in low resolution mode (*R* = 300) using a low-flow microconcentric nebulizer (flow rate was about 100 μL min^−1^). Instrument was tuned using a 1 ng g^−1^ multielement solution (Merck, Darmstadt, Germany). The optimization was carried out with respect to maximum uranium sensitivity and low UO^+^/U^+^ ratio.

For the Nd isotope ratio measurements NuPlasma™ (NU Instruments, Oxford, United Kingdom) double-focusing multi-collector inductively coupled plasma mass spectrometer (MC-ICP-MS) was used. Low mass resolution mode was used for all measurements. The sample introduction was done by a low-flow Teflon micro-concentric nebulizer in combination with a DSN-100 desolvation unit (NU Instruments, Oxford, United Kingdom). Instrument optimisation with respect to maximum sensitivity was carried out using a 100 ng g^−1^ Nd monoelemental solution (Alfa Aesar, Karlsruhe, Germany). The sensitivity was ~500 mV for ^143^Nd^+^ in 100 ng g^−1^ Nd standard solution.

The distribution of U and Th during the co-precipitation was followed by gamma spectrometric measurements using a well-type HPGe detector (GCW 2022 model) with ~20 % relative efficiency and a resolution of <1.7 keV at 185.6 keV (Canberra Industries Inc., USA). The measured spectra were evaluated using Genie 2000 v2.1 software. The measurement time varied between 600 and 5,400 s. All gamma spectrometric measurements were performed as relative measurements to the original starting material before and after the separation at fixed geometry.

All uncertainties quoted are given as expanded uncertainty using a coverage factor of *k* = 2 with last significant digits in parenthesis.

### Sample preparation

Approximately 0.5 g of samples were weighed into a Teflon Erlenmeyer and dissolved in 6 mL of 8 mol L^−1^ ultra-pure nitric acid while heating to 90 °C on a hot-plate for 12 h covered with a PE lid. After cooling to room temperature, the solution weights were measured.

About 3 mL of the stock solution, corresponding to about 200 mg of uranium, was transferred into a 50 mL polyethylene centrifuge vial. Ln, Th and U were precipitated as hydroxides (pH 12–14) with 40 % sodium hydroxide in the presence of 2 mg Fe(III) carrier. The supernatant, containing most of the alkali-soluble matrix elements (e.g. alkali metals) were carefully discarded after thorough centrifugation. Subsequently the precipitate was rinsed with high-purity water. Selective re-dissolution of uranium from the precipitate was done with 10 mL 1 % (NH_4_)_2_CO_3_ as uranium forms soluble di- and tri-carbonato complexes between pH 5–8 [15]. This step was repeated three to five times until clear solution was obtained assuring that U was removed from the sample to the highest extent possible. Representative aliquots of the supernatant were collected after each separation step in order to (i) control uranium decontamination and Th recovery factors by gamma spectrometric measurements parallel to the separation and (ii) use the achieved relatively pure uranium solution for other purposes (e.g. uranium isotope ratio measurement). The decontamination factor of the order of 10^2^–10^4^ achieved for U was sufficiently high to use extraction chromatography afterwards. The precipitate containing the Ln and Th was dissolved in 2 mL of 3 mol L^−1^ nitric acid to be in suitable form for further concentration. From this final solution 100 μL aliquots were taken for mass spectrometric measurements to evaluate recoveries and decontamination factors.

**Fig. 1:**
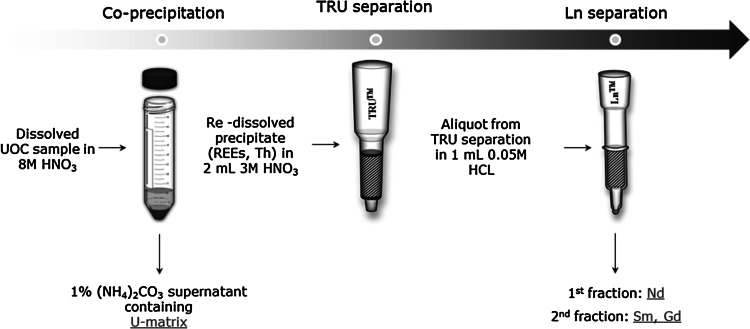
Scheme of the procedure developed

The Nd separation was performed in two steps: first a lanthanide group separation followed by the Nd separation. The Nd purification step is necessary for the removal of Sm, which interferes otherwise with the ICP-MS analysis. In the first step the lanthanide content of the sample aliquots was separated using extraction chromatography by the selective retention of trivalent lanthanides on the TRU™ resin in 3 mol L^−1^ nitric acid medium. In the second step, Ln resin was used in 0.05 mol L^−1^ HCl medium for the Nd separation. After Nd was stripped from the column with 0.2 mol L^−1^ HCl. This solution was evaporated to almost complete dryness and the residue was dissolved in nitric acid for mass spectrometric analysis. A method blank was processed through the entire dissolution and separation procedure parallel to the samples. The measured signal of the method blank was <2 % for all samples. The final Nd fractions were analysed by MC-ICP-MS. The simplified scheme of the entire separation procedure can be seen in Fig. 1. The detailed extraction chromatographic separation procedure can be found elsewhere [9, 16]. The method was validated by the measurement of reference material (Morille, Cetama), the recovery for the certified rare-earth elements (Sm, Eu, Gd, Dy) being better than 90 % [17].

## Results and discussion

**Fig. 2:**
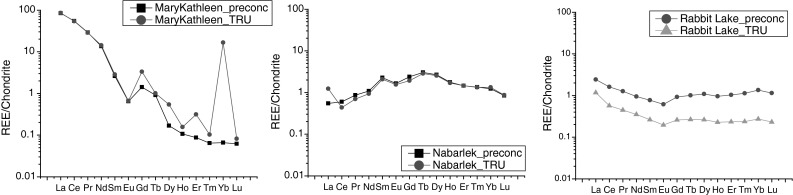
REE patterns of investigated uranium ore concentrate samples obtained with two different separation procedures: extraction chromatography with TRU resin (TRU) and in this work developed pre-concentration (preconc)

Six uranium ore concentrate samples were chosen to evaluate the capabilities of the method developed. Three of them (Rabbit Lake, Mary Kathleen and Nabarlek) were analysed to compare the REE pattern with and without pre-concentration step in order to verify that no interferences were introduced to samples by the used reagents. As one can see from Fig. 2, the REE patterns obtained with two different separation procedures agree well [16]. The high Yb level of Mary Kathleen sample is possibly related to isobaric interference if only TRU separation is applied. Our result of Mary Kathleen uranium ore concentrate sample is also in good agreement with the recently published work of Keegan et al. [18]. Note that REE patterns of the investigated uranium ore concentrate samples are presented after chondrite normalisation and in logarithmic scale [19].

The ^143^Nd/^144^Nd isotope ratio for three UOC samples (Rössing, Shirley Basin and CAN ESI) and BCR-2 geological standard was measured (Table 1). The measured ^143^Nd/^144^Nd value of the BCR-2 standard was 0.512598(78), which agrees with the certified value 0.512629(8) within uncertainty [20]. The Nd isotope ratio of Rössing sample had been measured previously using only extraction chromatography without pre-concentration and it resulted in a ^143^Nd/^144^Nd ratio of 0.51363(230) with high uncertainty. Comparing to our new result 0.51346(34), obtained with the improved sample pre-concentration procedure, one can see that the values are in good agreement.

**Table 1:** Measured ^143^Nd/^144^Nd isotope ratios in the investigated uranium ore concentrate samples

Sample	Country	Deposit type	c_Nd_ (ng g^−1^)	^143^Nd/^144^Nd_TRU_	^143^Nd/^144^Nd_preconc_
ESI	Canada	Phosphate	90	<DL	0.51225(9)
Rössing	Namibia	Intrusive	15	0.51363(230)	0.51346(34)
Shirley Basin	USA	Sandstone	8	<DL	0.51356(61)

All uncertainties quoted are given as expanded uncertainty using a coverage factor of *k* = 2 with last significant digits. ^143^Nd/^144^Nd_TRU_ corresponds to the results obtained by using only TRU separation, while ^143^Nd/^144^Nd_preconc_ are the results of the present method with preconcentration

Moreover, the uncertainty of the new result is almost an order of magnitude better. For Shirley Basin and CAN ESI the Nd isotopic composition couldn’t be measured previously when using only the TRU separation because of the Nd concentration being under detection limit. However, with the new sample preparation procedure precise results were obtained and they fit well in the previously found 0.510–0.515 range. With the improved Nd isotopic information and the lower uncertainties the uranium deposit type, and therefore the origin of an unknown nuclear material can be assessed with higher reliability.

## Conclusions

The sample amount in nuclear forensic investigations is of crucial importance, not just because the available sample amount is often limited as an evidence specimen, but also due to the need of relatively high amount of sample for the high precision elemental or isotopic analysis. Therefore, careful planning and sequencing of the measurements are required to perform comprehensive analyses. The proposed Fe(OH)_3_ co-precipitation in 1 % ammonium carbonate combines the effective pre-concentration of the trace-level constituents with the removal of the uranium matrix. The presented method is not just useful for the trace-level Nd isotope ratio analysis as demonstrated, but it is also a versatile and straightforward sample preparation procedure, which can be applied to pre-concentrate and separate other elements of interest, such as Th, Pu or Am from a single sample aliquot. These are just few examples of the promising potential of the newly developed pre-concentration procedure.
